# Leaf Morphological and Biochemical Responses of Three Potato (*Solanum tuberosum* L.) Cultivars to Drought Stress and Aphid (*Myzus persicae* Sulzer) Infestation

**DOI:** 10.3390/insects10120435

**Published:** 2019-12-04

**Authors:** Peter Quandahor, Chunyan Lin, Yuping Gou, Jeffrey A. Coulter, Changzhong Liu

**Affiliations:** 1 College of Plant Protection, Gansu Agricultural University, Lanzhou, No. 1 Yingmen Village, Anning District, Lanzhou 730070, China; quandooh@yahoo.com (P.Q.); linchunyan0905@163.com (C.L.); gouyp1988@163.com (Y.G.); 2 Department of Agronomy and Plant Genetics, University of Minnesota, St. Paul, MN 55108, USA; jeffcoulter@umn.edu

**Keywords:** potato, drought stress, aphid infestation, hydrogen peroxide, antioxidant enzyme activities

## Abstract

Drought stress on plants can cause cellular water deficits and influence the physiology of host plants, which alter the performance of insect pests. This study was conducted to determine the effect of drought and aphid (*Myzus persicae* Sulzer) infestation on three potato (*Solanum tuberosum* L.) genotypes under greenhouse conditions. A factorial experiment involving three potato genotypes, two levels of drought, and two levels of aphid infestation was conducted. The potato genotypes possessed different levels of tolerance to drought and are described as tolerant (*Qingshu 9*), moderately tolerant (*Longshu 3*), and sensitive (*Atlantic*). Sixty-day-old potato plants were infested with aphid nymphs and monitored for 20 d. There was a significant variety × drought × aphid interaction effect on the parameters measured. The genotype *Atlantic*, which is sensitive to drought, exhibited greater tolerance to aphid infestation under drought or no drought conditions than the other genotypes. This genotype also exhibited poor host acceptance and the aphid survival rate, colonization success, and average daily reproduction were low. *Qingshu 9*, which is tolerant to drought, was highly susceptible to aphid infestation and exhibited high host acceptance and greater aphid survival rate, colonization success, and average daily reproduction compared to the other genotypes. This study demonstrates that the biochemical and morphological traits that confer drought tolerance in potato do not necessarily confer aphid tolerance.

## 1. Introduction

The green peach aphid, *Myzus persicae* Sulzer, which has special adaptation features, such as the ability to adapt to the wide genetic variation of host plants, is an important crop pest that attacks more than 400 plant species across the globe [1]. The performance of *M. persicae* on plants exposed to drought seems to vary among plant species [2]. In *Arabidopsis thaliana*, the populations of *M. persicae* increased when the plants were exposed to drought stress [3]. However, in *Brassica oleracea* (cabbage), conflicting results were reported, as aphid abundance increased under drought in one experiment [4] but decreased under a similar condition in another experiment [5].

Drought is one of the major challenges facing crop production and about 40% of the world’s agricultural land is found in arid or semi-arid regions [6]. Drought stress on plants can cause cellular water deficits, membrane injury, reduced enzyme activity and crop yield, and even plant death [7]. By influencing the physiology of host plants, drought can alter the performance of insect pests. A number of studies have shown that increases in drought severity results in increasing populations of insect pests [8,9]. Plant morphological traits such as leaf shape, texture, and hairiness could influence the development of pest populations [10]. Leaf hairs are reported to affect the leaf boundary layer and serve as suitable habitats for mites [11]. The presence of leaf hairs in cotton (*Gossypium hirsutum*) increased the population of *Bemisia tabaci* on the plants [11,12]. However, the population of jassids (*Amrasca devastans*) on cotton varieties with hairy leaves decreased as compared with those that had smooth leaves [13].

Drought stress can damage the photosynthetic apparatus and decrease leaf chlorophyll content [14], transpiration rate, water content, and leaf angle [15]. These changes also affect aphid performance on host plants under drought stress [16]. Drought stress can cause carbon starvation, which subsequently changes host plant nutrition, palatability, and secondary metabolites that are capable of affecting aphid survival [15,17]. The purpose of stomata adaptation in response to water deficit is to enhance water use efficiency of the plant [18]. This occurs by altering stomata density and size [18]. The effect of water stress on stomata conductance depends on the severity and duration of the stress [19].

The initial signal events during defense responses in plants generally include the production of reactive oxygen species (ROS), malondialdehyde (MDA), and proline (Pro) [20,21,22,23]. Antioxidant enzymes such as superoxide dismutase (SOD), peroxidase (POD), and catalase (CAT) play a substantial role in defense reactions of host plants [22,23]. They can stimulate the transport of insect resistance signals during the defense response and induce the production of related compounds and enzymes in a cascade of reactions after insect invasion [24]. Potato (*Solanum tuberosum* L.) is the fourth most important food crop in the world after wheat (*Triticum aestivum*), rice (*Oryza sativa*), and maize (*Zea mays*) [25]. Potato production is increasing at a rate of 4.5 million tons per year, exceeding that of rice and wheat [26]. It has been estimated that by the year 2020, more than two billion people in Asia, Africa, and Latin America will depend on these crops for food, feed, or income [27]. The demand for potato is increasing rapidly in developing countries, which now account for more than half of the global harvest. Unfortunately, unfavorable weather conditions such as drought and the devastating effects of insect pests such as aphids are causing greater losses in arable crops including potato [6,25,28,29]. In Africa, Asia, and Latin America, for instance, yields could decline by 20% to 40% in the future if no effective adaptation measures are taken [25,30,31].

The identification of drought-tolerant cultivars as well as cultivars that possess some resistance to insect pests has become an important area of research [32,33]. Increasing aphid resistance and stress tolerance in potato has great potential to contribute to food and income security, mitigate poverty, and reduce farmers’ risk in vulnerable agricultural environments [29,33]. Several studies have reported the response of potato to drought stress [34,35,36], aphids to drought stress [37,38,39], and potato–aphid interactions [40,41]. However, to our knowledge, no study has determined the response of potato cultivars which have different levels of drought tolerance to aphid attacks. This research was based on the hypothesis that potato cultivars with different levels of tolerance to drought will respond differently to aphid attacks. The study was therefore conducted to determine the response of potato cultivars with different levels of drought tolerance to aphid infestation under greenhouse conditions.

## 2. Materials and Methods

### 2.1. Growth Conditions and Planting Materials

The experiment was conducted in a greenhouse (day temperature 25–35 °C, night temperature 18–22 °C, daytime relative humidity 45%–55%, light intensity 15,000–18,000 lux) at Gansu Agricultural University, Lanzhou, China, during the summer of 2019. Mini tubers of three potato genotypes were obtained from Gansu Haofeng Seed Company Limited, Lanzhou, China for the experiment. The three potato genotypes used for the experiment were known to possess different levels of tolerance to drought. These were *Qingshu 9* (tolerant)*, Longshu 3* (moderately tolerant), and *Atlantic* (sensitive). The tubers were sown in pots (12.5 cm diameter, 9.5 cm deep) filled with 2 kg of loamy soil. Each pot was supplied with one tuber and the soil was watered periodically to keep it moist. All pots were watered uniformly until 40 d after sowing, when the drought treatment began. The pots were kept free of weeds by regularly hand-picking the weeds that emerged.

### 2.2. Aphid Culture

Adult females of *Myzus persicae*, collected from the potato experimental farm of Gansu Agricultural University, Lanzhou, China, were reared in the laboratory on potato seedlings under a light:dark 16:8 h photocycle at 19 ± 1 °C. The aphid culture was maintained for six months before it was used for the experiment.

### 2.3. Experimental Design and Treatments

The experiment was a 3 × 2 × 2 factorial involving three potato genotypes, *Qingshu 9* (QS9)*, Longshu 3* (L3), and *Atlantic* (DXY), two levels of drought stress (drought and no drought stress), and two levels of aphid infestation (aphid infestation and no aphid infestation). The treatments were arranged in a split–split plot design with three replications. The three genotypes were assigned to main plots and each of these main plots was split into two for the drought and no drought treatments. Each of these split-plots was further split into two for the aphid and no aphid treatments. In all, 216 pots were used for the experiment and each experimental unit consisted of six pots with one plant per pot. The plants under no drought treatment were watered adequately to maintain the soil moisture at field capacity. Whereas in the drought-stressed plants the water content was allowed to withdraw progressively for 20 d and then maintained with 30% of the field capacity throughout the experiment. To maintain the levels of water through time in each treatment, the pots were weighed every 5 d, and the water volume for the corresponding field capacity was supplied. Soil moisture was also measured daily with a Delta-T Theta Probe ML2 (Delta-T Devices, Cambridge, UK) to keep all pots’ consistency.

### 2.4. Determination of Aphid Performance

Six shoots of each genotype were infested with 20 nymph aphids, respectively, with the two drought conditions (*n* = 20 in each treatment). Host acceptance was measured by counting the number of aphid nymphs which remained on plants at 3 d after infestation. Aphid survival rate was measured by counting the number of nymphs that remained and developed into adults to reproduce at 16 d after infestation. The aphid survival rate (%) was calculated as the number of aphids that survived to reproduce/total number of nymphs × 100. Colonization success was assessed by counting the number of adult aphids and the number of nymphs produced up to the 20th day after infestation. The colonization success was then estimated as the number of nymphs + ln (number of adults + 0.001), which gives a balanced weight to the number of nymphs and adults produced on a plant 20 d after infestation [42]. Average daily reproduction was determined as the number of nymphs divided by the number of days for the period of infestation.

### 2.5. Aphid Water Content

Aphid water content was determined as described by Guo et al. [43]. Adult aphids from each plant were collected at 20 d after infestation and weighed immediately, dried at 60 °C for 24 h, and then weighed again. Water content was determined by deducting the dry weight from the fresh weight of each aphid. Aphid water content (AWC) was calculated as:

(1) $$\mathrm{AWC}=\frac{\mathrm{FW}-\mathrm{DW}}{\mathrm{DW}}\times 100\%$$

### 2.6. Leaf Relative Water Content (RWC)

The RWC of leaves was determined as described by Barrs and Weatherley [44]. The four youngest, fully expanded leaves were removed from six shoots of each genotype and the fresh weight (FW) was determined immediately. The leaves were immersed in distilled water for 6 h, then removed, and the adhering water was blotted with tissue paper before weighing to obtain turgor weight (TW). The dry weight (DW) was measured after drying the leaves at 70 °C in an oven for 24 h. The relative water content (RWC) was calculated as follows:

(2) $$\mathrm{RWC}=\frac{\mathrm{FW}-\mathrm{DW}}{\mathrm{TW}-\mathrm{DW}}\times 100\%$$

### 2.7. Relative Plant Biomass

The shoots of 16 plants per treatment were harvested at the end of the experiment to determine the impact of drought and aphids on plant biomass. Plant material was oven-dried to constant weight at 80 °C for 72 h to determine the mean dry weight (DW) per plant. The relative plant biomass (RPB) was calculated as follows:

(3) $$\mathrm{RPB}=\frac{\mathrm{Shoot}\;\mathrm{biomass}\;\mathrm{under}\;\mathrm{stress}}{\mathrm{Shoot}\;\mathrm{biomass}\;\mathrm{under}\;\mathrm{no}\;\mathrm{stress}}\times 100\%$$

### 2.8. Chlorophyll Content, Net Photosynthesis, Transpiration Rate, and Leave Angle

Chlorophyll content, net photosynthesis, transpiration rate, and leave angle of six shoots of each genotype were measured on the fifth to tenth terminal of mature leaves from the base of the shoot at 30 d after infestation. Chlorophyll content (ChlSPAD value) was estimated using a portable chlorophyll meter (CCM-200, Opti-Sciences, Tyngsboro, MA, USA). Light-saturated net photosynthesis was measured with a portable infrared gas analyzer (LI-6200, LI-COR, Lincoln, NE, USA). Transpiration rate was measured with a portable porometer (LI-1600, LI-COR, Lincoln, NE, USA). Leaf angle was measured using an Accupar LP-80 ceptometer (Decagon Devices, NE Hopkins Court, Pullman, WA, USA).

### 2.9. Stomata and Leaf Hair Analysis

Three leaves from six shoots of each genotype were collected at 20 d after infestation, for stomata and leaf hair analysis. Scanning of the leaf to obtain digital images was done using a scanning electron microscope following a standard method [45]. Samples were fixed in 3% glutaraldehyde buffered with 0.1 M phosphate buffer at room temperature for 2 h. Samples were then washed with 0.1 M phosphate buffer and dipped in 1% osmium tetroxide in 0.1 M phosphate buffer (pH 7.2) for 2 h at room temperature in a light proof container. Samples were again washed in 0.1 M phosphate buffer (pH 7.2), dehydrated in classified ethanol solutions in water (30%, 50%, 70%, 80%, 90%, 96%, and 100% ethanol solution) for 5 to 15 min, and dried. This process involved the replacement of liquid in the cells with gas and creates a completely dry specimen with negligible or no cellular distortion. The specimens were mounted on an aluminum stub using double-sided sticky carbon tape. Gold coating was made using a gold sputter coater that coats the mounted specimens in gold before they go into the SEM. Samples were then analyzed directly in SEM using Smart SEM TM software (Carl Zeiss, EVO18, Welwyn Garden City, UK). Leaf hairiness was assessed by counting the number of trichomes on a 15 mm^2^ square area per leaf. Stomata number was determined by counting the stomata within the 15 mm^2^ square area.

### 2.10. Determination of Proline (Pro) Content

Proline content in the leaf samples was determined after extraction at room temperature with 3% of 5-sulfosalicylic acid solution as described by Bates et al. [46] using a standard curve. Free proline content was expressed as μmol g^−1^ FW of leaves [47].

### 2.11. Determination Hydrogen Peroxide (H_2_O_2_) Content

Hydrogen peroxide content was determined as described by Mostofa and Fujita [48]. Frozen leaf samples were homogenized and centrifuged, and the supernatant was collected and reacted with TiCl_4_ and NH_4_OH. After the second centrifuge, the supernatant was discarded and the precipitate washed recurrently with cold acetone until it turned colorless. The washed precipitate was dissolved in 20 mL 2 M H_2_SO_4_, and the absorbance measured at 415 nm against a blank. Standard H_2_O_2_ was treated with TiCl_4_ and subjected to the similar process.

### 2.12. Determination of Malondialdehyde (MDA) Content

The content of MDA was measured as described by Liu et al. [49]. In brief, potato leave samples were homogenized in 5% (*w*/*v*) trichloroacetic acid and reacted with an equal volume of 0.67% (*w*/*v*) thiobarbituric acid in a water bath for 30 min. After cooling, the mixture was centrifuged and the supernatant used to measure the absorbance at 532 nm and corrected for nonspecific turbidity by subtracting the absorbance at 600 and 450 nm.

### 2.13. Determination of Antioxidant Enzyme Activities in Leaf Tissue

The SOD, CAT, and POD activities were determined using leaf tissues that were stored at −80 °C. Briefly, about 0.5 g of leaf tissues were ground in liquid nitrogen and then homogenized in 5 mL of 0.1 M phosphate buffer (pH 7.5) containing 0.5 mM Ethylene Diamine Tetra-acetic Acid (EDTA). The homogenates were centrifuged at 12,000× *g* for 15 min at 4 °C, and the supernatant was aliquoted for SOD (EC 1.15.1.1), CAT (EC 1.11.1.6), and POD (EC 1.11.1.7) activity assays. The supernatants were then used in determining the enzymatic activities. The SOD activity was measured following a standard procedure [50]. The POD activity was estimated at 470 nm as described by Chance and Maehly [51], while CAT activity was measured following the method of Nakano and Asada [52].

### 2.14. Statistical Analysis

Data were analyzed by the analysis of variance using SPSS statistics software (Version 19.0 for Windows, SPSS, Chicago, IL, USA). Treatment means were separated using Duncan’s multiple range test *(p* *<* 0.05). The results are presented as mean ± SD.

## 3. Results

### 3.1. Aphid Performance

**Figure 1:**
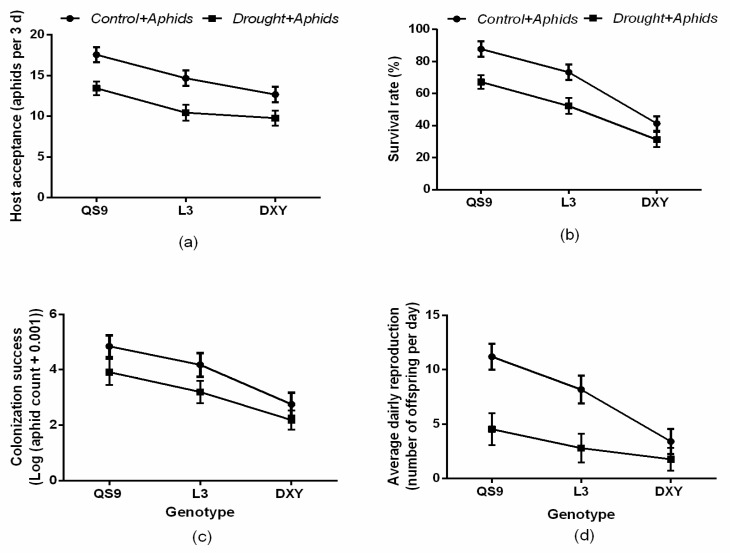
Changes in (**a**) host acceptance, (**b**) survival rate, (**c**) colonization success, and (**d**) average daily reproduction in three potato cultivars grown under well-watered or drought conditions with and without peach aphid infestation. Data represent the mean ± SD of three replicates. QS9: *Qingshu 9*; L3: *Longshu* 3; DXY: *Atlantic.*

There was a significant (*p <* 0.01) genotype × drought interaction effect on aphid survival rate, colonization success, and average daily reproduction (Figure 1). However, host acceptance was not affected (*p* = 0.06) by the genotype × drought interaction. Generally, host acceptance, survival, colonization success, and average daily reproduction of aphids were higher on the drought-free plants. Aphid survival rate on plants under no drought (87.7%) and those under drought (67.2%) was highest on QS9 and least on DXY (41.3% and 31.2%, respectively) (Figure 1a–d). Although host acceptance was not affected (*p* = 0.06) by the genotype × drought interaction, it was highest on QS9 under no drought (87.7%) and under drought (67.2%) and least on DXY (63.3 and 48.8%, respectively) (Figure 1a). Colonization success of aphids on plants under no drought and on those exposed to drought was highest on QS9 (24.2 and 19.5%, respectively) and least on DXY plants (13.7 and 10.9%, respectively) (Figure 1c). Relative to all water treatments, colonization success increased by 24.2% and 19.5%, 72.8% and 20.8%, and 18.4% and 10.9% on QS9, L3, and DXY, respectively. Under drought conditions, average daily reproduction was highest (29.3%) on QS9 and least (19.3%) on DXY. Moreover, average daily reproduction was 51.8% and 29.3%, 40.8% and 22.3%, and 24.8% and 19.3% on QS9, L3, and DXY under no drought and drought, respectively (Figure 1d). Thus, among the genotypes, QS9 had the greatest host acceptance, aphid survival, colonization success, and average daily reproduction with or without drought.

### 3.2. Aphid Water Content and Leaf Relative Water Content

**Figure 2:**
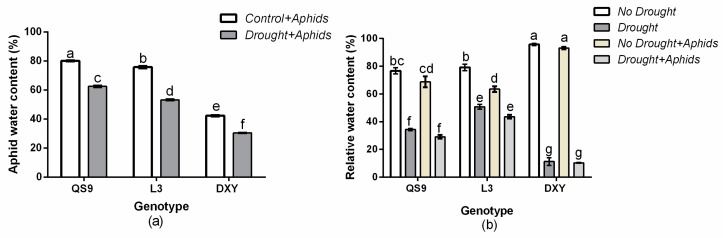
Changes in (**a**) aphids water content and (**b**) relative water content in three potato cultivars grown under well-watered or drought conditions with and without peach aphid infestation. Data represent the mean ± SD of three replicates. Lower case letters indicate statistically significant differences between genotypes within the same water treatment and aphid treatment by LSD test (*p* < 0.05).

The genotype × drought interaction significantly (*p* < 0.01) affected aphid water content (Figure 2a). Aphid water content on well-watered plants was higher than in plants under drought across the genotypes. In well-watered plants, aphid water content was 80.1%, 75.7%, and 24.8% for QS9, L3, and DXY, respectively. However, in plants under drought stress, aphid water content was 62.5%, 53.2%, and 19.3%, respectively. Aphid water content was higher for aphids on QS9 and lower for aphids on DXY. Moreover, the analysis of leaf relative water content showed a significant *p* < 0.001) genotype × drought × aphid interaction effect (Figure 2b). Generally, leaf relative water content of all genotypes significantly (*p* < 0.01) decreased under drought stress as compared to the respective control plants. However, aphid infestation significantly (*p* < 0.001) decreased leaf relative water content of QS9 and L3 but did not affect DXY with or without aphid infestation. In comparison with control plants, leaf relative water content of QS9, L3, and DXY was 50.8%, 34.3%, and 11.2% under drought stress and 63.5%, 68.8%, and 93.2% under aphid infestation. The DXY cultivar had higher (93.2%) leaf relative water content under aphid infestation but lower (11.2%) leaf relative water content under drought stress compared with the other genotypes.

### 3.3. Plant Growth Response to Drought Stress and Aphids Treatments

**Figure 3:**
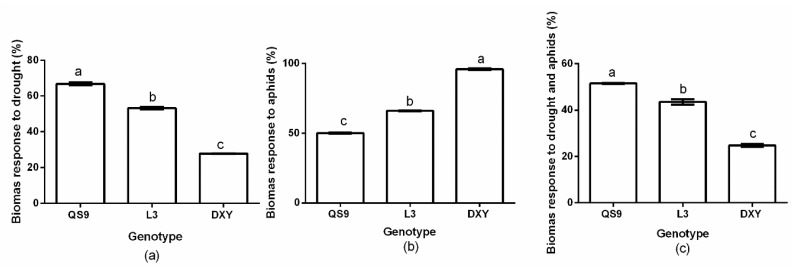
Changes in biomass response to (**a**) drought, (**b**) aphids, and (**c**) drought and aphids in three potato cultivars grown under well-watered or drought conditions with and without peach aphid infestation. Data represent the mean ± SD of three replicates. Lower case letters indicate statistically significant differences between genotypes within the same water treatment and aphid treatment by LSD test (*p* < 0.05).

Biomass accumulation of the potato genotypes was significantly (*p* < 0.01) affected by drought stress and aphid treatment. The QS9 genotypes had the highest biomass (66.8%) under drought stress conditions (Figure 3a). However, under aphid infestation, the highest (95.8%) biomass occurred on DXY plants (Figure 3b). Under the influence of both drought and aphid infestation, QS9 plants had the highest biomass (51.5%, Figure 3c). Plant biomass was higher in DXY (95.8%), but lower in the QS9 (66.1%), under aphid infestation. Based on these results, DXY and QS9 were considered the most resistant and susceptible cultivars to aphids, respectively.

### 3.4. Correlation Analysis

Correlations between aphid performance and plant indexes of the potato genotypes under drought stress showed some significantly positive and negative correlations (Table 1). For instance, RWC was positively correlated with survival rate (SR), host acceptance (HA), leaf hair density (LHD), plant biomass (PB), and stomata size (SS). Moreover, colonization success (CS) was positively correlated with SR and HA, but negatively correlated with SS. Survival rate was positively correlated with HA, PB, and SS. Host acceptance was positively correlated with PB and SS, but negatively correlated with LHD. Leaf hair density was positively correlated with PB and SS. There was also a positive correlation between PB and SS.

### 3.5. Genotypic Variation of Physiological Response to Drought Stress and Aphids Treatments

**Figure 4:**
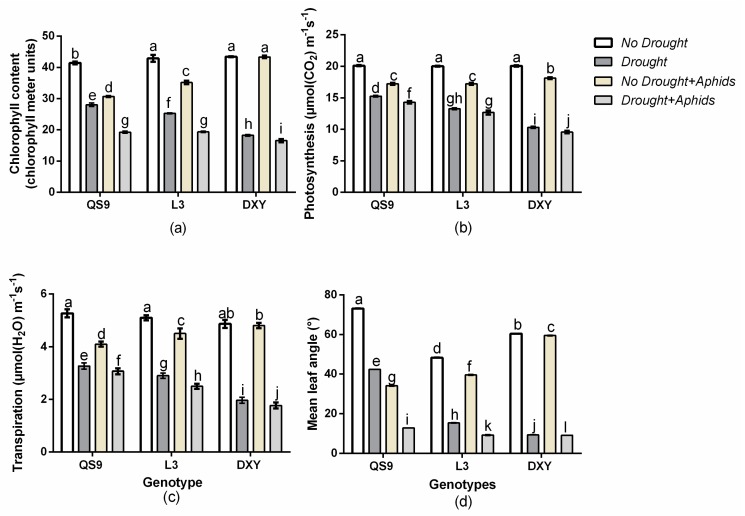
Changes in (**a**) chlorophyll content, (**b**) photosynthesis, (**c**) transpiration, and (**d**) mean leave angle in three potato cultivars grown under well-watered or drought conditions with and without peach aphid infestation. Data represent the mean ± SD of three replicates. Lower case letters indicate statistically significant differences between genotypes within the same water treatment and aphid treatment by LSD test (*p* < 0.05).

Leaf chlorophyll content, net photosynthesis, transpiration rate, and mean leaf angle are important physiological and morphological traits that can indicate the level of stress in plants. There was a significant (*p* < 0.01) genotype × drought × aphid interaction effect on leaf chlorophyll content, net photosynthesis, transpiration rate, and mean leaf angle. Drought significantly (*p* < 0.01) reduced leaf chlorophyll content, net photosynthesis, transpiration rate, and mean leaf angle of all genotypes relative to their corresponding control plants (Figure 4). Drought stress decreased leaf chlorophyll content of QS9, L3, and DXY by 32.3%, 41.0%, and 57.9%, respectively, in respect to the control plants. Under aphid infestation, leaf chlorophyll content of QS9, L3, and DXY also decreased by 25.9%, 17.9%, and 0.1% relative to the control plants. The least decrease in leaf chlorophyll content under drought stress (32.3%) and aphid infestation (0.1%) was observed in QS9 and DXY, respectively (Figure 4a). Drought stress also decreased net photosynthesis of QS9, L3, and DXY by 47.4%, 75.6%, and 76.3% relative to the control plants. Under aphid stress, net photosynthesis of QS9, L3, and DXY decreased by 42.1%, 33.5%, and 8.9%, respectively (Figure 4b). Moreover, drought stress also significantly decreased the transpiration rate of QS9, L3, and DXY by 20.9%, 44.4%, and 67.5% in respect to the control plants. Under aphid stress, the transpiration rate of QS9, L3, and DXY decreased by 13.9%, 7.1%, and 1.6% compared to the control plants (Figure 4c). Under drought stress, the mean leaf angle of QS9, L3, and DXY significantly (*p <* 0.01) decreased by 41.9%, 68.1%, and 69.8% respectively. Mean leaf angle of QS9, L3, and DXY also decreased by 53.2%, 18.1%, and 1.2% in respect to the control plants under aphid stress (Figure 4d). Comparatively, DXY had the lowest leaf chlorophyll content, net photosynthesis, transpiration rate, and mean leaf angle among genotypes under drought stress. However, under aphid infestation, the lowest leaf chlorophyll content, net photosynthesis, transpiration rate, and mean leaf angle among cultivars were observed on QS9 (Figure 4a–d).

### 3.6. Changes in Leaf Hair Density and Number of Potato Cultivars under Drought and Aphid Treatments

**Figure 5:**
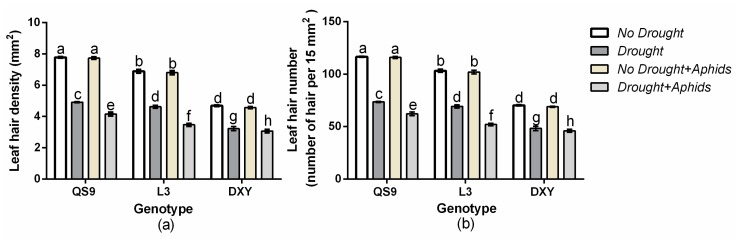
Changes in (**a**) leaf hair density and (**b**) number in three potato cultivars grown under well-watered or drought conditions with and without peach aphid infestation. Data represent the mean ± SD of three replicates. Lower case letters indicate statistically significant differences between genotypes within the same water treatment and aphid treatment by LSD test (*p* < 0.05).

There was a significant (*p <* 0.01) genotype × drought × aphid effect on leaf hair density and number. Although leaf hair density and number were similar for the three genotypes, under drought stress, DXY had the least leaf hair density and number relative to the control (Figure 5). Conversely, aphid infestation decreased leave hair density and number of QS9 and L3, but not DXY, regardless of aphid status.

### 3.7. Changes in Leaf Stomata Density, Number, and Size of Potato Genotypes under Drought and Aphid Treatments

**Figure 6:**
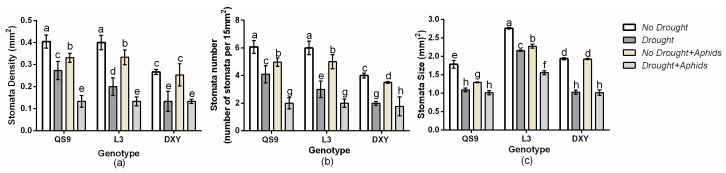
Changes in (**a**) stomata density, (**b**) number, and (**c**) size in three potato cultivars grown under well-watered or drought conditions with and without peach aphid infestation. Data represent the mean ± SD of three replicates. Lower case letters indicate statistically significant differences between genotypes within the same water treatment and aphid treatment by LSD test (*p* < 0.05).

There was a significant (*p <* 0.01) genotype × drought × aphid effect on leaf stomata density, number, and size. A similar trend of leaf hair density and number was also observed in stomata density, number, and size among the genotypes. Under drought stress, DXY had lower stomata density, number, and size than QS9 and L3 in respect to the controls (Figure 6). Aphid infestation decreased stomata density, number, and size of QS9 and L3, but did not affect those of DXY, regardless of the aphid status.

### 3.8. Effect of Drought Stress and Aphid Interaction on MDA, H_2_O_2_, and Proline Contents

**Figure 7:**
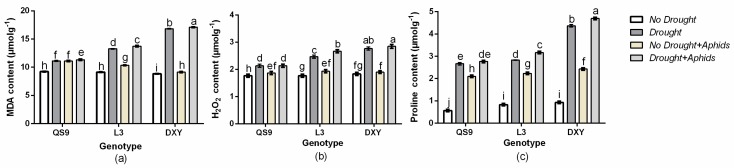
Changes in (**a**) malondialdehyde (MDA), (**b**) H_2_O_2_, and (**c**), and proline content in three potato cultivars grown under well-watered or drought conditions with and without peach aphid infestation. Data represent the mean ± SD of three replicates. Lower case letters indicate statistically significant differences between genotypes within the same water treatment and aphid treatment by LSD test (*p* < 0.05).

Drought usually causes injury at the cellular level by increasing the production of ROS and accumulation of MDA. The accumulation of Pro is usually considered to be an adaptive response against environmental stresses [44]. Meanwhile, an increase of MDA reflects impairment to the structural integrity of cell membranes caused by oxidative stress such as that derived from drought [45]. To associate these functional attributes with drought and aphid tolerance exhibited by potato genotypes, MDA, H_2_O_2_, and Pro contents were analyzed. Accordingly, MDA, H_2_O_2_, and Pro contents in the various genotypes were different from each other under the well-watered condition (Figure 7a–c). Drought stress and aphid infestation resulted in an overall increase in MDA, H_2_O_2_, and Pro content in all genotypes compared to those under the control condition. In comparison with control plants, drought stress increased MDA content of QS9, L3, and DXY by 20.6%, 31.1%, and 45.1%, respectively. Under aphid stress, the accumulation of MDA of QS9, L3, and DXY genotypes was also increased by 17.0%, 11.6%, and 3.2%, respectively. The H_2_O_2_ content in QS9, L3, and DXY increased by 26.6%, 28.1%, and 32.5% under drought and 16.4%, 11.25, and 4.1% under aphid stress, respectively. The content of Pro in QS9, L3, and DXY similarly increased by 62.5%, 70.75, and 78.7% under drought stress and 69.7%, 60.6%, and 26.3% under aphid infestation, respectively. The DXY plants accumulated more MDA, H_2_O_2_, and Pro under drought stress compared to the other genotypes, whereas the least accumulation of MDA, H_2_O_2_, and Pro among cultivars was recorded in QS9. In contrast, there was a significant reduction in MDA, H_2_O_2_, and Pro content in DXY under aphid infestation compared with the other genotypes. The greatest MDA, H_2_O_2_, and Pro content occurred in QS9 under aphid infestation.

### 3.9. Effect of Drought Stress and Aphid Interaction on Antioxidant Enzymes Activities

**Figure 8:**
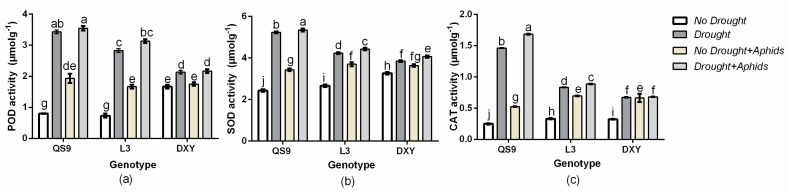
Changes in (**a**) peroxidase (POD), (**b**) superoxide dismutase (SOD), and (**c**) catalase (CAT) activities in three potato cultivars grown under well-watered or drought conditions with and without peach aphid infestation. Data represent the mean ± SD of three replicates. Lower case letters indicate statistically significant differences between genotypes within the same water treatment and aphid treatment by LSD test (*p* < 0.05).

In this study, changes in ROS scavenging activities such as POD, SOD, and CAT were investigated to assess whether differences can be associated with resistance to drought stress and aphid infestation. The results show that POD, SOD, and CAT activities increased in all genotypes under drought and aphid stress compared to their respective controls (Figure 8a–c). Under drought stress, POD activity in QS9, L3, and DXY increased by 78.6%, 58.6%, and 41.1%, respectively, and by 58.6%, 41.1%, and 37.1% under aphid stress, respectively. The SOD activity in QS9, L3, and DXY also increased by 49.1%, 41.15, and 33.0% under drought stress and 29.1%, 27.95, and 2% under aphid stress, respectively. The CAT activity in QS9, L3, and DXY also increased by 77.9%, 62.8%, and 52.2% under drought stress and 52.6%, 52.2%, and 36.6% under aphid stress, respectively. The POD, SOD, and CAT activities were higher in QS9 than the other cultivars under drought stress and aphid infestation, whereas the lowest POD, SOD, and CAT activities occurred in DXY.

## 4. Discussion

The availability of water is critical for plant growth and their resistance to insect pests [7,53]. Khan et al. [4] reported that sap sucking insect pests, such aphids, thrive better on water-stressed plants than on normal plants. In contrast, Simpson et al. [5] reported that these insect pests thrive better on plants grown without water stress. Notably, only some field experiments support the concept that aphid density increases on drought-stressed plants; the experimentally imposed water stress, nevertheless, often adversely affects aphid performance [2]. In the current study, higher aphid performance was observed on the well-watered plants compared with the drought-stressed plants across all genotypes. Drought stress decreased all aphid performance indexes, including host acceptance, aphid survival, colonization success, and average daily reproduction, across genotypes. The decreased performance of the aphids on drought-stressed plants observed in this experiment is similar to the findings of Mewis et al. [3], who reported that populations of *M. persicae* increased on *Brassica oleracea* under no drought stress. Other studies showed that aphid performance increased on plants under drought stress [54,55]. Although drought stress negatively affected aphid performance across genotypes, the QS9 genotype was the most susceptible host because the aphids thrived better on it. The drought-sensitive genotype (DXY) showed great resistance to aphids under drought or no drought conditions. Thus, the aphid performance indexes were low regardless of the water treatment applied. It is reported that the effect of drought stress on herbivore abundance may depend on the type of plant, type of damage, herbivore feeding guild and species, and stress intensity and duration [56,57]. Drought-induced carbon starvation may change host plant nutrition, palatability, and resistance with respect to herbivores [18]. Host plant chemical composition can be modified as a result of drought stress [37,58,59], which can affect aphid performance positively [43,60] or negatively [37,61], or in some cases have no effect [62,63]. Moreover, plants are known to contain secondary metabolites that are capable of affecting aphid survival [57]. This suggests that tolerance of potato genotypes to drought could influence their resistance to aphids. Thus, the resistance to aphids observed in the DXY genotype could be attributed to both the decreased water content and possible increases in the levels of secondary metabolites. The inherent ability of plants to resist aphid infestation and the water status of the growing medium both play significant roles in influencing aphid performance [19]. The DXY genotype, which exhibited resistance to aphids could be promoted in areas where aphids pose a challenge, as proposed by Xu et al. [33].

Plants respond and adapt to drought stress through the induction of various morphological and physiological responses [64,65]. Many physiological factors may be involved in drought stress injury [66]. Drought stress can damage the photosynthetic apparatus and decrease leaf chlorophyll content [14], as well as transpiration rate, water content, and leaf angle [15]. These changes also affect aphid performance on host plants under drought stress [16]. In the current study, drought stress decreased relative water content, leaf chlorophyll content, net photosynthesis, transpiration rate, and mean leaf angle of all genotypes. Although these indexes were decreased, they were relatively higher in the DXY genotype, under aphid infestation. Moreover, the DXY plants under aphid infestation had a higher biomass and the aphids on these plants had lower water content. This gives an indication of the poor performance of aphids on the DXY genotype. These results are similar to the findings of Guo et al. [43], who reported contrasting tolerance to aphid infestation among *Medicago truncatula* cultivars. We speculate that under drought stress, high water content in the host plant is crucial for aphid performance. Accordingly, plant responses to water stress, including changes in the plant’s resistance and water status, should be examined when considering the effect of water stress on plant–insect interactions. In the current study, drought stress decreased the water content of all genotypes, which adversely affected host acceptance, survival rate, colonization success, and average daily reproduction of the green peach aphid on all cultivars under drought stress, compared with their respective control plants. The higher water content of the tolerant genotype (QS9) improved the performance of the green peach aphids under drought conditions. In contrast, the high water loss of the sensitive cultivar (DXY) dramatically decreased their performance. These observations were supported by positive correlations of RWC with SR and HA across the three genotypes under drought stress and aphid infestation. Furthermore, HA positively correlated with PB and SS, but negatively correlated with LHD. This suggests that higher PB and SS have the tendency to support aphid performance. On the other hand, higher leaf LHD could interfere with aphid feeding, thereby decreasing their performance. High RWC, PB, and SS of plants could play an important role in the performance of green peach aphids under drought stress.

The presence of leaf hairs results in increases in the populations of *Bemisia tabaci* on cotton [12]. However, the populations of jassids on cotton with hairy leaves decreased as compared with those cultivars that had smooth leaves [13]. In this experiment, the DXY genotype, which had a smooth leaf surface and was without leaf hairs, was more resistant to aphid infestation compared to the other genotypes. This genotype had the least number of aphids that survived on it. Leaves with smooth, hairless surfaces may be more prone to having the boundary layer disrupted by wind currents than hairy leaves, thereby making them less suitable for the development of pests [11,67]. This might have contributed to the aphid tolerance of the DXY plants. Casson and Gray [68] reported that short-term responses to unfavorable conditions cause plants to alter their stomata aperture, but when such conditions persist, the stomata density or size may be affected. The results of our experiment show that drought stress and aphid infestation decreased stomata density, number, and size of all cultivars. The DXY plants had the least stomata density, number, and size compared with the other genotypes when exposed to drought stress. This gives an indication of their poor tolerance to drought stress. Under moderate stress stomata number increases, but it decreases under severe water stress [19]. Drought stress also has an effect on stomata size, with smaller stomata being observed in plants under drought stress [18]. In this study, aphid infestation decreased stomata density, number, and size in QS9 and L3 plants, but did not affect them in DXY plants, suggesting that the DXY genotype is more resistant to aphids than the other genotypes.

The early signal events during defense responses in plants usually include the production of higher contents of ROS, MDA, and Pro [20,22]. Our results show that drought stress and aphid infestation increased H_2_O_2_, MDA, and Pro contents in all genotypes compared to the respective control plants. However, the increase in H_2_O_2_ and MDA contents was least in QS9 under drought stress and in DXY under aphid stress. The contents of H_2_O_2_ and MDA in DXY plants under aphid stress did not differ significantly from those without aphids, probably because of its tolerance to aphids. The increase in Pro content observed in all cultivars under drought stress did not improve aphid performance, probably because Pro acted as a mediator of osmotic adjustment and might have acted in this case as a stress-related signal more than as a nutrition substrate for aphids [69]. The drought-sensitive genotype, DXY, which exhibited tolerance to aphids, accumulated a higher amount of Pro compared to the other genotypes.

To avoid or alleviate cell damage caused by ROS, plants stimulate their antioxidant enzyme system, and these protective enzymes are closely related to plant stress resistance. The SOD activity provides the first line of defense against membrane lipid peroxidation induced by ROS [24]. In our study, SOD, CAT, and POD activities of all genotypes increased under drought stress and aphid infestation, but the rate of increase varied among genotypes. The activities of POD, SOD, and CAT were highest in QS9 plants under drought stress and aphid infestation, while the least POD, SOD, and CAT activity occurred in DXY plants. The activity of antioxidant enzymes in plants is believed to be an indication of their level of tolerance to stress [24,70]. Although QS9 plants were more tolerant to drought stress compared to the other genotypes, they exhibited greater susceptibility to the aphids with high host acceptance as well as greater survival rate, colonization success, and average daily reproduction of the aphids. These results show that SOD and CAT activities play important roles in the tolerance of QS9 plants to drought, but this does not protect the plants against aphid stress. The POD, SOD, and CAT activities under aphid stress were lower in DXY plants, even though they exhibited greater tolerance to aphids with or without drought stress. This was demonstrated by greater biomass accumulation in the DXY plants. In addition, host acceptance, survival rate, colonization success, and average daily reproduction of aphids on this genotype were low. This was probably because the DXY genotype contains compounds that reduce feeding by the peach aphid. Thus, the results of our experiment suggest that the mechanism of tolerance to drought in potato plants differs from the mechanism that may confer aphid tolerance.

## 5. Conclusions

The results of this study show that the drought-sensitive genotype, DXY, exhibited greater tolerance to peach aphids under drought or no drought conditions. This was demonstrated by poor aphid performance and higher biomass accumulation of this genotype. However, the drought-tolerant genotype, QS9, was highly susceptible to the peach aphid. This was shown by the high aphid performance and low biomass accumulation. Furthermore, the extreme water loss of the sensitive genotype, DXY, dramatically decreased the performance of the green peach aphid under drought stress. The drought-tolerant genotype exhibited high water content on which the green peach aphid is able to absorb from the xylem sap, thereby improving the performance of the green peach aphid. Resistance of the DXY genotype to the peach aphid may also be due to the presence of toxic compounds in the plants, since it exhibited poor host acceptance. Thus, the DXY genotype can be utilized to increase potato yield in areas where peach aphids are a major constraint. This study demonstrates that the biochemical and morphological traits that confer drought tolerance in potato do not necessarily confer aphid tolerance. Further studies to ascertain the presence and levels of compounds that could inhibit feeding by peach aphids could advance our knowledge on the response of these genotypes to peach aphids.

## Figures and Tables

**Table 1:** Pearson’s correlation coefficients for correlations between aphid performance and host plant indexes of three potato genotypes under drought stress and aphid infestation.

	RWC	AWC	ADR	CS	SR	HA	LHD	PB	SS
RWC	1	0.206	0.217	0.359	0.545 *	0.588 *	0.599 **	0.886 **	0.596 **
AWC		1	0.151	0.23	0.317	0.248	−0.433	0.184	−0.442
ADR			1	0.78	−0.496	0.464	−0.529	0.429	−0.433
CS				1	0.765 **	0.812 **	−0.231	0.171	−0.560 *
SR					1	0.983 **	−0.448	0.514 *	0.495 *
HA						1	−0.468 *	0.513 *	0.527 *
LHD							1	0.752 **	0.475 **
PB								1	0.541 **
SS									1

* Significant at *p* < 0.01; ** significant at *p* < 0.05; RWC: relative water content; AWC: aphid water content; ADR: average daily reproduction; CS: colonization success; SR: survival rate; HA: host acceptance; LHD: leaf hair density; PB: plant biomass; SS: stomata size.
